# Radiosynthesis of ^11^C-phenytoin Using a DEGDEE Solvent for Clinical PET Studies

**DOI:** 10.22038/aojnmb.2018.10846

**Published:** 2018

**Authors:** Yasukazu Kanai, Yoshinori Miyake, Eku Shimosegawa, Jun Hatazawa

**Affiliations:** 1 Department of Molecular Imaging in Medicine, Osaka University Graduate School of Medicine, Osaka, Japan; 2 Department of Tracer Kinetics and Nuclear Medicine, Osaka University Graduate School of Medicine, Osaka, Japan

**Keywords:** PET, Radiopharmacy, Radiosynthesis, ^11^C-phenytoin

## Abstract

**Objective(s):**

Phenytoin is an antiepileptic drug that is used worldwide. The whole-body pharmacokinetics of this drug have been extensively studied using ^11^C-phenytoin in small animals. However, because of the limited production amounts that are presently available, clinical ^11^C-phenytoin PET studies to examine the pharmacokinetics of phenytoin in humans have not yet been performed. We aimed to establish a new synthesis method to produce large amounts of ^11^C-phenytoin to conduct human studies.

**Methods:**

[^11^C] methane was produced using an in-house cyclotron by the ^14^N (p, α) ^11^C nuclear reaction of 5 % of hydrogen containing 95 % of nitrogen gas. About 30 GBq of ^11^C-methane was then transferred to a homogenization cell containing Fe_2_O_3_ powder mixed with Fe granules heated at 320 ^0^C to yield ^11^C-phosgene. Xylene, 1,4-dioxane, and diethylene glycol diethyl ether (DEGDEE) were investigated as possible reaction solvents.

**Results:**

The ratio of ^11^C-phenytoin radioactivity to the total ^11^C radioactivity in the reaction vessel (reaction efficiency) was 7.5% for xylene, 11% for 1,4-dioxane, and 37% for DEGDEE. The synthesis time was within 45 min from the end of bombardment until obtaining the final product. The radioactivity produced was more than 4.1 GBq in 10 mL of saline at the end of synthesis. The specific activity of the product ranged from 1.7 to 2.2 GBq/μmol. The quality of the [^11^C] phenytoin injection passed all criteria required for clinical use.

**Conclusion:**

The use of DEGDEE as a solvent enabled the production of a large amount of ^11^C-phenytoin sufficient to enable PET studies examining the human pharmacokinetics of phenytoin.

## Introduction

Phenytoin is a well-characterized and widely used anti-epileptic drug (1-3). Phenytoin is a voltage-dependent Na^+^ channel blocker that suppresses the excitation of neurons. In the past, the distribution of ^11^C-labeled phenytoin and its derivatives has been studied using positron emission tomography (PET) in animals and humans. Meldrum et al. synthesized ^11^C-hydantoin analogs, including ^11^C-phenytoin, using ^11^C- HCN (4). In their study, the radioactivity of the ^11^C-phenytoin was 74 MBq at the time of injection, and the total synthesis time was 106 min. Stavchansky et al. and Emaran et al. produced ^11^C-hydantoin analogs from ^11^C-HCN using different precursors (5, 6). Roeda et al. synthesized ^11^C-phenytoin from ^11^C-phosgene (7). In these previous studies, however, the difficulty of producing a stable and large amount of ^11^C-phenytoin restricted the clinical use of ^11^C-phenytoin for measuring the whole-body distribution. We previously reported an ^11^C-phenytoin kinetic study in small animals where only small amounts of ^11^C-phenytoin (5 MBq) were used (8). In the previous report, we did not describe the details of synthesis methods. Baron et al. reported ^11^C-phenytoin accumulation in the human brain (9). Although ^11^C-phenytoin PET was expected to be a useful tool for pharmacokinetic studies of phenytoin and a biomarker of voltage-dependent Na^+^ channel distribution and density in humans, other researchers have not pursued this potential because of the difficult radiosynthesis method.

The purpose of the present study was to establish a synthesis method for ^11^C-phenytoin suitable for clinical use. A large amount of radioactivity (at least 370 MBq) at the time of injection and a high level of safety were required. In this study, we investigated various reaction conditions for ^11^C-phenytoin, especially the selection of a reaction solvent.

## Methods

***Chemicals***

The precursor of ^11^C-phenytoin, 2-amino-2,2-diphenylacetamide, was provided by Dainippon Seiyaku Co., Ltd. (Osaka, Japan). Phenytoin was purchased from Wako Pure Chemical Co., Ltd. (Osaka, Japan). Other chemicals and solvents were purchased as follows: iron granules (10–40 mesh, 99.999%) from Aldrich Chemical Co., Milwaukee, WI; iron (III) oxide powder (98.0%) and chlorine gas (99.999%) from Asahi Denka Kogyo K.K., Tokyo, Japan; and antimony powder (99.8%) from Merck KGaA., Darmstadt, Germany. These reagents were used without further purification.

***Synthesis of*** ^11^ ***C-phosgene***

**Figure 1:**
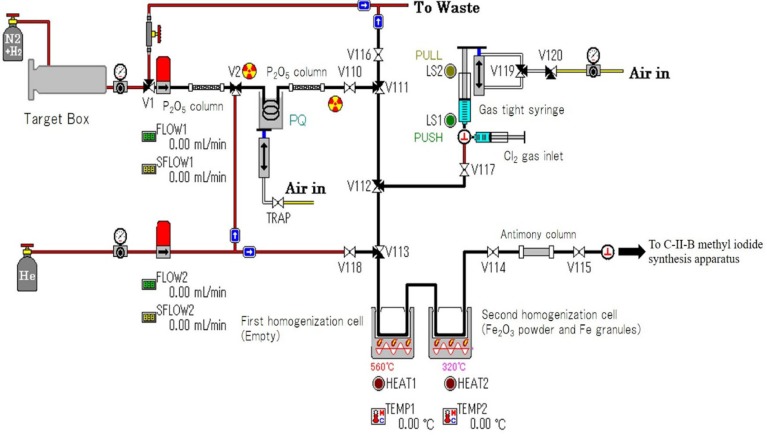
Diagram of an automated synthesis apparatus for ^11^C-phenytoin

^11^C-phosgene was produced according to a method previously reported by Nishijima et al. (10). Briefly, ^11^C-methane was produced using an in-house cyclotron (CYPRIS-HM18, Sumitomo Heavy Industries, Tokyo, Japan) by a ^14^N(p, α)^11^C nuclear reaction on 5 % of hydrogen containing 95 % of nitrogen gas. The irradiation conditions were a 10–15 μA proton beam for 40–60 min at 18 MeV. An automated synthesis apparatus (CUPID, Sumitomo Heavy Industries) was used for the radiolabeling of ^11^C-phosgene (Figure 1). About 40 GBq of ^11^C-methane produced in the target box was trapped and condensed in a stainless U-tube (4 mm; I.D., 150 mm) immersed in liquid nitrogen and filled with Porapak Q (80–100 mesh; Waters, Milford, MA). The stainless U-tube was then warmed to room temperature. The ^11^C methane within the stainless U-tube was transferred by helium flow to the first homogenization cell (glass–Teflon gas-tight syringe). 2 ml of chlorine gas was added to the first homogenization cell by gas tight syringe. Then the first homogenization cell was heated at 560 ^0^C. In this step, ^11^C-methane was converted to ^11^C carbon tetrachloride (^11^C-CCl_4_). ^11^C-CCl_4_ was transferred to the second homogenization cell filled with Fe_2_O_3_ powder and Fe granules (1.5 g; Fe_2_O_3_ powder/Fe granules, 1/28, w/w). The second homogenization cell was heated at 320 ^0^C to yield ^11^C phosgene. Finally, the ^11^C-phosgene was passed through an antimony column to remove chlorine.

***Investigation of reaction solvents***

Xylene, 1,4-dioxane, and diethylene glycol diethyl ether (DEGDEE) were investigated as possible reaction solvents. The precursor of ^11^C-phenytoin was dissolved in each solvent. ^11^C-phosgene was then bubbled in a reaction vessel containing each solvent. The reaction vessel was then heated to a temperature near boiling (140 ^0^C for xylene, 100 ^0^C for 1,4-dioxane, and 180 ^0^C for DEGDEE) for 10 min.

***Synthesis of*** ^11^ ***C-phenytoin injection***

**Figure 2.**
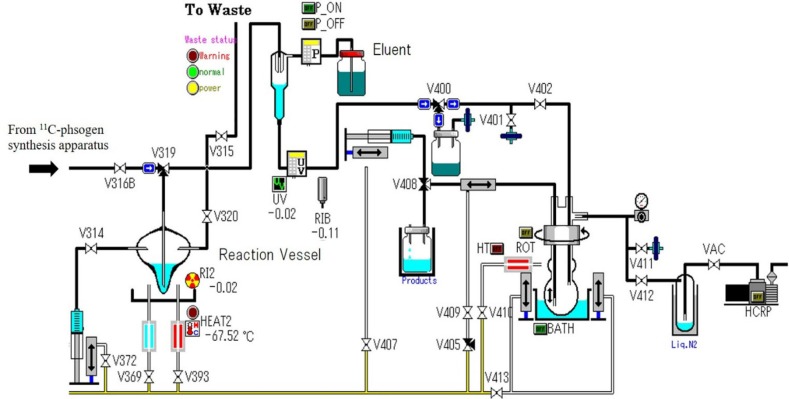
Diagram of C-II-B methyl iodide synthesis apparatus

**Figure 3:**
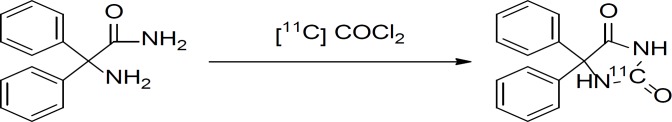
Synthesis route of ^11^C-phenytoin

About 30 GBq of ^11^C-phosgene was introduced to a reaction vessel containing 4 mg of 2-amino-2,2-diphenylacetamide in 2.0 mL of DEGDEE and set in a C-II-B methyl iodide synthesis apparatus (Figure 2 and Figure 3), with 200 mL/min of helium gas. Then, the reaction mixture was heated to 180 ^0^C for 10 min. The reaction was quenched by the addition of 2.5 % ammonia solution and ethanol mixture (25/75, v/v). The reaction mixture was transferred to an HPLC system. The HPLC separation conditions were as follows: a YMC-pack polymer C18 column (10 mm × 250 mm; YMC, Kyoto, Japan), a separation eluent consisting of 2.5 % ammonia solution and ethanol (25/75, v/v), and a flow rate of 2.0 mL/min. The retention time of ^11^C-phenytoin was 8 min.

After HPLC separation, 100 mL of the ^11^C-phenytoin fraction was collected in a recovery flask. The solvent was removed by evaporation. The residue was resolved using saline. The final product was sterilized by filtration using a Millex-GV filter (Merck Millipore, Darmstadt, Germany). 

***Quality control for*** ^11^ ***C-phenytoin injections***

The items examined for quality control were pH, color and particles in injection solution, radionuclide impurities, radiochemical purity, physical half-life, endotoxin test, sterilization test, and residual amount of ammonium ion, ethanol, and DEGDEE in the injection solution. Radiochemical purity and specific activity of ^11^C-phenytoin were measured by analytical HPLC. Analytical HPLC was performed using a Polymer C18 column and 50 mM Na_3_PO_4_ + 5 mM sodium dodecyl sulfate/acetonitrile (60/40, v/v). We confirmed that the major radioactivity is ^11^C-phenytoin with analytical HPLC. The retention time is corresponding with that of non-radioactive standard. Specific activity of ^11^C-phenytoin is calculated from total amount of phenytoin. Amount of phenytoin was determined from UV absorption calibration curve of non-radioactive phenytoin analysis. The ammonia concentration was measured using Fuji Dry Chem 100 and Fuji Dry Chem slide NH_3_-PII (Fuji Film Medical, Tokyo, Japan). The ethanol and DEGDEE concentrations were measured using gas chromatography (GC-14B; Shimadzu, Kyoto, Japan). For ethanol, a TSG-1 15 % SHINCARBON A 60/80 3.2 × 3.3, 100 mm column was used (Shimadzu, Kyoto, Japan). The column temperature was 90 ^0^C. A Flame Ionization Detector was used. The detector temperature was 180 ^0^C. The carrier gas was nitrogen, and the flow rate was 30 mL/min. Under these conditions, ethanol was detected at 4 min. For DEGDEE, a G-300 40 m × 1.2 mm column was used (Chemicals Evaluation and Research Institute, Japan, Tokyo, Japan). The column temperature was 130 ^0^C. A Flame Ionization Detector was used. The detector temperature was 180 ^0^C. The carrier gas was helium, and the flow rate was 30 mL/min. Under these conditions, DEGDEE was detected at 4.5 min.

The quality control of the ^11^C-phenytoin product was validated according to the criteria of the Safety Control Committee for Short-lived Radiopharmaceuticals, Osaka University Hospital.

## Results

***Comparison among reaction solvents***

The ratio of ^11^C-phenytoin radioactivity to the total ^11^C radioactivity in the reaction vessel (reaction efficiency) was 7.5 % for xylene, 11.0 % for 1,4-dioxane, and 37.0 % for DEGDEE. Because DEGDEE had the highest reaction efficiency among these three solvents, the synthesis and quality control of ^11^C-phenytoin was studied using DEGDEE.

***Synthesis of*** ^11^ ***C-phenytoin solution***

The synthesis time was within 45 min from the end of bombardment to the final product. The radioactivity produced was more than 4.1 GBq at the end of synthesis (Table 1).

***Quality control for*** ^11^ ***C-phenytoin solution***

**Figure 4:**
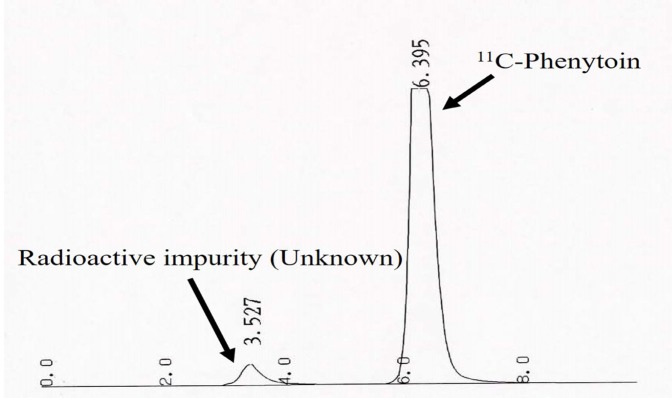
Radio-HPLC chart of ^11^C-phentoin for radiochemical purity analysis

^11^C-phenytoin was produced three times. The total procedure for quality control was completed within 20 min. The results of the quality control and the criteria of the Safety Control Committee for Short-lived Radiopharmaceuticals, Osaka University Hospital, are shown in Table 1. All the samples were clear and colorless by visual inspection. No particles were visually detected. No bacterial colonies were detected two weeks after the study. The values for specific activity, pH, radionuclide impurities, radiochemical purity, physical half-life, and amount of ammonium ion, ethanol, and DEGDEE were within the ranges of the quality control criteria. The HPLC chromatogram of radiochemical purity measurement is shown in Figure 4.

## Discussion

Roeda et al. reported the use of xylene for the synthesis of ^11^C-phenytoin (7). Their study indicated that the use of xylene as a solvent resulted in a high reaction efficiency of around 70 %. However, in the present study, the reaction efficiency was only 7.5 %. In addition, xylene must be removed before HPLC purification because of its high lipophilicity. Because of the low reaction efficiency and long synthesis time, we concluded that xylene was not an appropriate solvent for ^11^C-phenytoin production for use in clinical PET studies.

**Table 1:** Results of ^11^C-phenytoin synthesis and quality control criteria

	**Run 1**	**Run 2**	**Run 3**	**Criterion value**
**Radioactivity (EOS)**	6.2 GBq	7.9 GBq	4.1 GBq	--------------------
**Radioactivity for Injection **	3.1 GBq	3.5 GBq	2.0 GBq	
**Specific activity**	1.9 GBq/μmol	2.2 GBq/μmol	1.7 GBq/μmol	>1.0 GBq/μmol
**pH**	6.3–6.9	6.3–6.9	6.3–6.9	5.0–8.0
**Color**	Clear and colorless	Clear and colorless	Clear and colorless	Clear and colorless
**Particles**	None	None	None	None
**Radionuclide impurities**	511 keV only	511 keV only	511 keV only	511 keV only
**Radiochemical purity**	>95%	>95%	>95%	>95%
**Half-life**	20.3 min	20.0 min	20.4 min	19-21 min
**Endotoxin test**	Pass	Pass	Pass	<0.25 EU/mL
**Sterilization test**	Pass	Pass	Pass	Pass
**Ammonium ion**	91 μg/dL	17 μg/dL	46 μg/dL	200 μg/dL
**Ethanol**	<10 ppm	<10 ppm	<10 ppm	<2000 ppm
**DEGDEE**	<10 ppm	<10 ppm	<10 ppm	<100 ppm

We selected the water-soluble solvents 1,4-dioxane and DEGDEE to avoid the need for a solvent removal procedure prior to HPLC purification. The synthesis time was within 45 min when these solvents were used. The reaction efficiencies were 10 % and 37 % for 1,4-dioxane and DEGDEE, respectively. Therefore, we selected DEGDEE as a solvent for the production of large amounts of ^11^C-phenytoin for use in clinical PET studies.

The European Union and USA authorities have recommended the use of human PET studies with labeled candidate compounds during the early phase of new drug development (EU, USA) (11, 12). Our previous study of ^11^C-donepezil indicated that the whole-body absorption, distribution, metabolism, and excretion of clinically prescribed medicines can be evaluated in humans using PET (13). In such studies, the mass dose of the tracer was limited to 100 mg or less with adequate radioactivity for imaging according to the recommendations by EU and USA authorities (EU, USA). In the present study, the specific activity of ^11^C-phenytoin after quality control was 1.9, 2.2, and 1.7 GBq/μmol. These activities corresponded to 750, 870, and 670 MBq/100 mg at the time of injection. In recent clinical PET studies with currently available scanners using ^11^C labeled tracers such as ^11^C-methionine, the injection dose is approximately 3 MBq/kg, or around 200 MBq/body (14). Therefore, the current synthesis method for ^11^C-phenytoin provides sufficient radioactivity with a tracer amount of less than 100 mg. Optimization of the labeling procedure, reagents, and quality control should be further investigated in the future.

## Conclusion

The use of DEGDEE as a solvent enabled the production of a large amount of ^11^C-phenytoin solution with a high specific activity sufficient to enable PET studies examining the human pharmacokinetics of phenytoin.
